# Aldosterone Induces DNA Damage and Activation of Nrf2 Mainly in Tubuli of Mouse Kidneys

**DOI:** 10.3390/ijms21134679

**Published:** 2020-06-30

**Authors:** Ronja Balhorn, Christina Hartmann, Nicole Schupp

**Affiliations:** Institute of Toxicology, University of Düsseldorf, 40225 Düsseldorf, Germany; Balhorn.Ronja@hhu.de (R.B.); Christina.Hartmann.2@hhu.de (C.H.)

**Keywords:** aldosterone, Nrf2, kidney damage, chronic kidney disease, distal tubuli

## Abstract

Hypertensive patients have an increased risk of developing chronic kidney disease (CKD). Many of these patients have increased levels of the blood pressure regulating mineralocorticoid aldosterone. As a protection against aldosterone-induced damage, kidney cells can upregulate key regulators of the antioxidant defense, such as nuclear factor-erythroid-2-related factor 2 (Nrf2). In the present study aldosterone-induced kidney damage and Nrf2 activation in kidney cells of mice treated with three different concentrations of aldosterone for 4 weeks was localized. Increased albumin and neutrophil gelatinase-associated lipocalin (NGAL) in urine revealed an impaired kidney function of the aldosterone-infused mice. Localization of aldosterone-induced oxidative damage (in the form of DNA lesions) in specific kidney cells showed an increase in proximal tubuli and to an even greater extend in distal tubuli. Phosphorylated Nrf2 was increased in distal tubule cells after aldosterone-infusion. Nrf2 activation in proximal tubuli or in glomeruli after aldosterone-treatment could not be observed. Nrf2 target genes and proteins analyzed, paradoxically, showed a downregulation in the whole kidney. Aldosterone-treated mice exhibited an increased kidney injury and DNA damage in distal and proximal tubuli. Nrf2 seemed only to be specifically activated in distal tubule cells, where we also detected the highest amount of oxidative damage.

## 1. Introduction

Not many therapeutic options exist to decelerate progression of CKD. Standard therapy at the moment is administration of inhibitors of the renin–angiotensin–aldosterone system (RAAS). Angiotensin converting enzyme inhibitors (ACEi) and angiotensin II receptor blockers (ARB) exert antihypertensive as well as anti-inflammatory and anti-fibrotic effects. The latter are explained by reduction of angiotensin II levels and subsequently aldosterone levels, which both are known to activate the pro-inflammatory transcription factor nuclear factor ‘kappa-light-chain-enhancer’ of activated B-cells (NF-κB), as well as the pro-fibrotic protein transforming growth factor-β (TGF-β). Aldosterone receptor antagonists reduce proteinuria and attenuate progressive renal disease, pointing to an important impact of aldosterone [1,2].

Inflammatory stimuli like interleukin-6 (IL-6), tumor necrosis factor-α (TNF-α), or also aldosterone increase the production of reactive oxygen species (ROS) [2,3]. Oxidative stress is increased in CKD, documented by elevated levels of markers of lipid-, protein-, and DNA-oxidation in the serum of CKD patients compared to healthy controls [4,5,6] and endogenous antioxidant defense is reduced in CKD [7,8]. Although studies with antioxidant interventions were promising in animal models, results of clinical studies are controversial. While, for example, the antioxidant *N*-acetylcysteine could ameliorate several endpoints of disease complication, like loss of glomerular filtration or cardiac events, no beneficial effects were observed on all-cause mortality [9].

Since exogenous supplementation with antioxidants seems inefficient, a therapeutic option for the treatment of chronic kidney disease could be activators of the endogenous oxidative defense. Here, the transcription factor Nrf2, which controls primary defense mechanisms against oxidative stress as well as phase 2 metabolic enzymes is a promising target. Nrf2 activation was found to be suppressed in CKD [10,11], Nrf2 polymorphisms have an impact on survival of CKD patients [12], and animals lacking Nrf2 show pathological changes in kidney morphology, loss of kidney function [13], as well as an increased susceptibility to nephrotoxic compounds [14]. Studies of Nrf2 enhancers of the triterpenoid class (bardoxolone methyl especially) in experimental kidney disease [14,15] as well as in first human studies were initially promising [16,17]. The subsequent phase 3 clinical trial in diabetic patients with CKD [18] had to be terminated due to serious adverse cardiovascular events [19]. Despite this setback, further research is warranted, since (1) Nrf2 activation still is one of the most promising therapeutic approaches in CKD and (2) there exists a variety of other substance classes with Nrf2-activating potential, which might not have similar severe side effects as bardoxolone methyl. The lesson of the triterpenoid bardoxolone methyl, nevertheless, illustrates quite dramatically that further insight into the beneficial and deleterious effects of pharmacological approaches activating Nrf2 is needed.

We could show before that aldosterone or its precursor deoxycorticosterone acetate provoke detectable kidney damage within 4–6 weeks of treatment, like an increase in albuminuria and morphological changes, which were independent of the blood pressure increase and reduced by antioxidants [20,21,22]. Aldosterone also induced oxidative stress in the kidneys of the animals used in these studies, resulting, among other damages, in DNA damage [20,21,22]. Further, we observed an aldosterone-induced Nrf2 activation in kidneys of rats which was not sufficient to protect from the adverse aldosterone effects, while additional treatment with a Nrf2 activator could protect the kidneys from oxidative stress and pathological tissue damage [21].

In the present study, to find out which cells in the kidney react to kidney damage with a change in Nrf2 activation, we treated mice with three different aldosterone concentrations to induce a loss of kidney function. In the kidneys of these mice, the localization of activated Nrf2 as well as the expression of Nrf2 targets was studied. To be able to co-localize the activated Nrf2 with oxidative damage on the tissue, we chose to use DNA damage as a marker of this damage, since as a nuclear marker it is easier to locate than oxidative damage markers of proteins or lipids.

## 2. Results

### 2.1. Blood Pressure Changes and Clinical Characteristics

**Figure 1:**
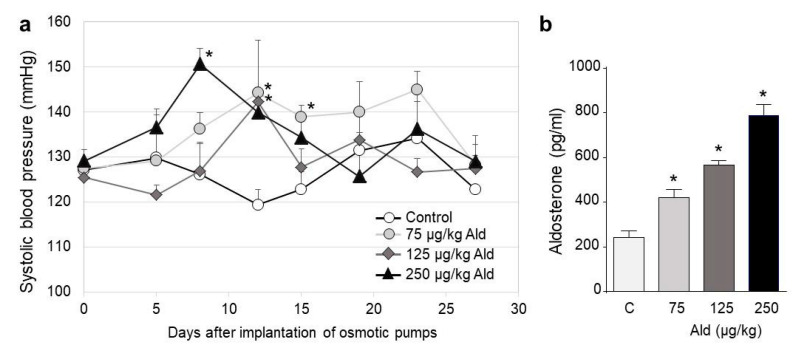
Blood pressure changes and aldosterone levels. (**a**) Development of systolic blood pressure in the control and aldosterone treatment groups. The blood pressure was measured at seven time points after acclimatization to the measurement procedure. The first time point represents the initial blood pressure before the implantation of osmotic pumps. The blood pressure was measured twice a week. (**b**) Aldosterone levels in the blood after 28 days of treatment. Ald: aldosterone. Data are shown as mean + SEM. * *p* ≤ 0.05 vs. C: control group.

The three chosen doses of aldosterone, 75, 125, and 250 µg/kg × d did not significantly increase the blood pressure compared to the control over the whole treatment period (Figure 1a). There was a tendential increase in the first 2 weeks of the treatment in all aldosterone-infused groups. The last 2 weeks of treatment seemed to be affected on the one hand by an adaptation in the aldosterone-treated animals and by a non-significant increase in the blood pressure of the control group. Quantification of the aldosterone levels in the serum of the four mouse groups showed a significant increase in all dose groups (Figure 1b). 

The body weight of the aldosterone-treated animals was not changed compared to the control group (Table 1). Kidney to body weight ratio was significantly higher in all dose groups, while heart to body weight ratio was significantly increased by the two higher aldosterone doses.

### 2.2. Renal Function, Histopathological Changes of the Kidney and Systemic Inflammatory Cytokines

Drinking volume and diuresis showed a trend to higher volumes in the aldosterone-treated animals, with the lowest dose group reaching a significantly higher drinking volume compared to the control. While there was no significant change in creatinine clearance, all three aldosterone groups displayed a massive albuminuria and had significantly increased concentrations of the kidney injury marker NGAL in urine (Table 1).

**Figure 2:**
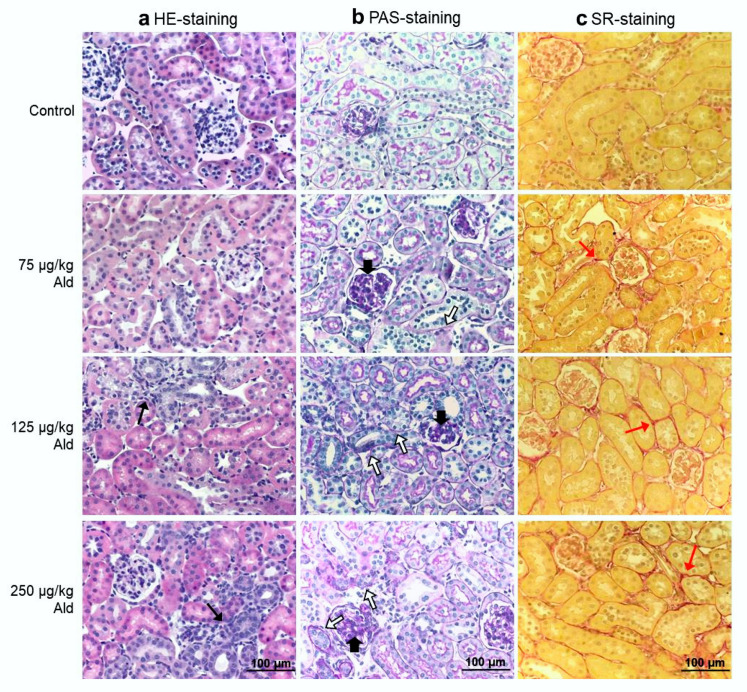
Histopathological changes in kidneys of control and aldosterone-treated mice. (**a**) Representative pictures of hematoxylin and eosin (HE) stained tissue for visualization of inflammatory regions. (**b**) Representative pictures of periodic acid–Schiff (PAS) stained kidney sections for visualization of basal membrane atrophy. (**c**) Representative pictures of Sirius red (SR) stained tissue for visualization of collagen deposition (red). Black narrow arrows: migrated leukocytes as a marker of beginning inflammation. Black broad arrows: examples of sclerotic tissue in glomeruli. White filled arrows: examples of atrophic basal membranes. Red arrows: collagen deposition. Ald: aldosterone.

Pathological changes of tubuli, mainly characterized by collagen deposition and atrophy of the basal membranes, were significantly increased in all dose groups, while areas of inflammation were only significantly increased by the two higher aldosterone doses (Table 2 and Figure 2). Aldosterone also led to glomerular damage. Significant signs of glomerular sclerosis (GSI, Table 2) were found in all treatment groups, while increased widening of capillaries (MSI, Table 2) was only significant in the high dose group.

Quantification of circling cytokines in serum revealed no consistent and even no dose-dependent effect of the three aldosterone doses (Table S1). The only significant effect was observed for IL-17A in the middle aldosterone dose.

### 2.3. Oxidative Stress and DNA Damage Induced by Aldosterone in the Kidney

**Figure 3:**
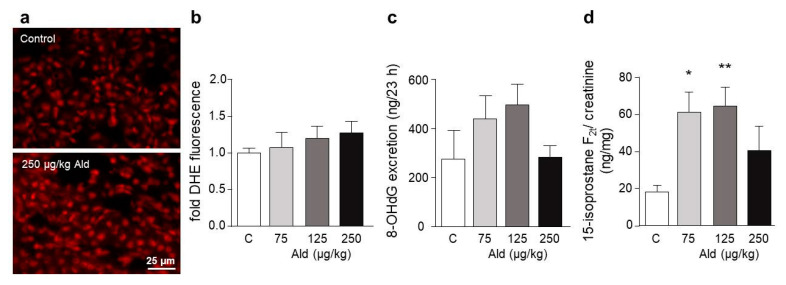
Reactive oxygen species (ROS) in kidneys of aldosterone-infused mice. For the visualization of ROS, cryosections of kidneys were incubated with the ROS-sensitive fluorescent dye dihydroethidium (DHE). (**a**) Representative pictures of DHE stained kidney sections. (**b**) Quantification of ROS production by measuring the mean grey value of nuclei within 10 visual fields with Image J. (**c**) Amount of the excised oxidized nucleobase 8-OHdG excreted in urine. (**d**) Amount of the lipid peroxidation product 15-isoprostane F_2t_ related to urinary creatinine. 8-OHdG: 8-hydroxy-2′-deoxyguanosine, Ald: aldosterone. Data are shown as mean + SEM, *n* = 4–5. * *p* ≤ 0.05, ** *p* < 0.01 vs. C: control group.

ROS production on cryosections of the kidney visualized with the redox-sensitive dye dihydroethidium was tendentially increased in all three aldosterone doses (Figure 3). 8-OHdG as a urinary marker of oxidative damage to nucleobases was also only tendentially increased, but 15-isoprostane F_2t_ as a marker of oxidative damage to lipids was significantly higher in the low and middle dose groups and tendentially increased in the high dose group.

**Figure 4:**
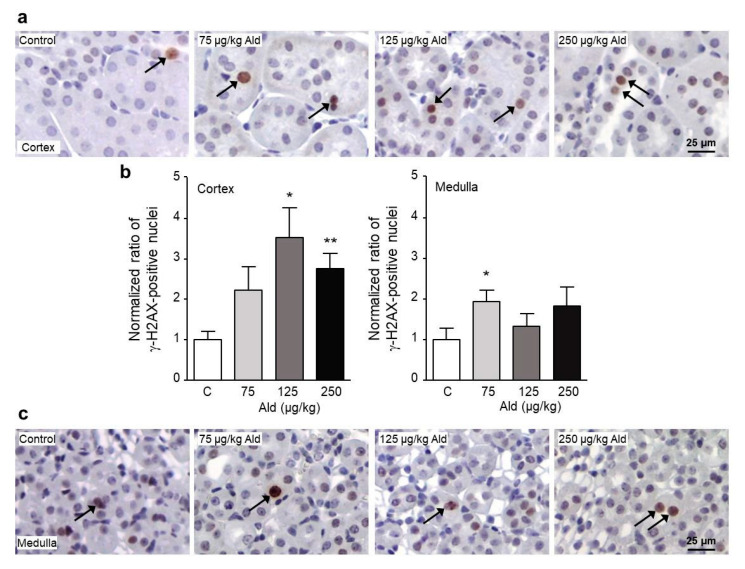
DNA damage caused by aldosterone infusion. Paraffin-embedded kidney sections were stained with an antibody against γ-H2AX, a marker of structural DNA damage. Staining of γ-H2AX in cortex (**a**) and medulla (**c**). (**b**) Quantification as ratio of γ-H2AX-positive stained nuclei normalized to the control. For the quantification of γ-H2AX-positive nuclei, 10 visual fields of cortical and five visual fields of medullary kidney sections were analyzed per animal via Image J. Examples of positive stained nuclei are marked with black arrows. γ-H2AX: phosphorylated histone 2AX, Ald: aldosterone. Data are shown as mean + SEM, *n* = 5. * *p* ≤ 0.05, ** *p* < 0.01 vs. C: control group.

Oxidatively modified guanosine, 8-oxodG, was also analyzed on tissue (Figure S1). A non-significant increase of the number of 8-oxodG-positive cells was observed in the low and high dose groups in both cortex and medulla. Oxidative DNA damage in the form of DNA double strand breaks detected with the help of an antibody against the DNA damage surrogate marker γ-H2AX was increased in the kidney cortex by all aldosterone doses, significantly by the middle and the highest dose (Figure 4a,b). While there was also a slight increase of γ-H2AX-positive cells seen in the kidney medulla, this was only significant for the lowest dose (Figure 4b,c). No reduction of the expression of DNA repair enzymes like Ogg1, Brca1, or Apex1 could be detected in the aldosterone-treated mouse kidneys (Figure S2). On the contrary, a significantly increased protein expression of the DNA damage response related proteins PARP and PCNA could be shown (Figure S2). The detected three to four-fold increase in PCNA-positive nuclei could also be an indication of a higher proliferation rate in kidneys in response to the induced damage.

**Figure 5:**
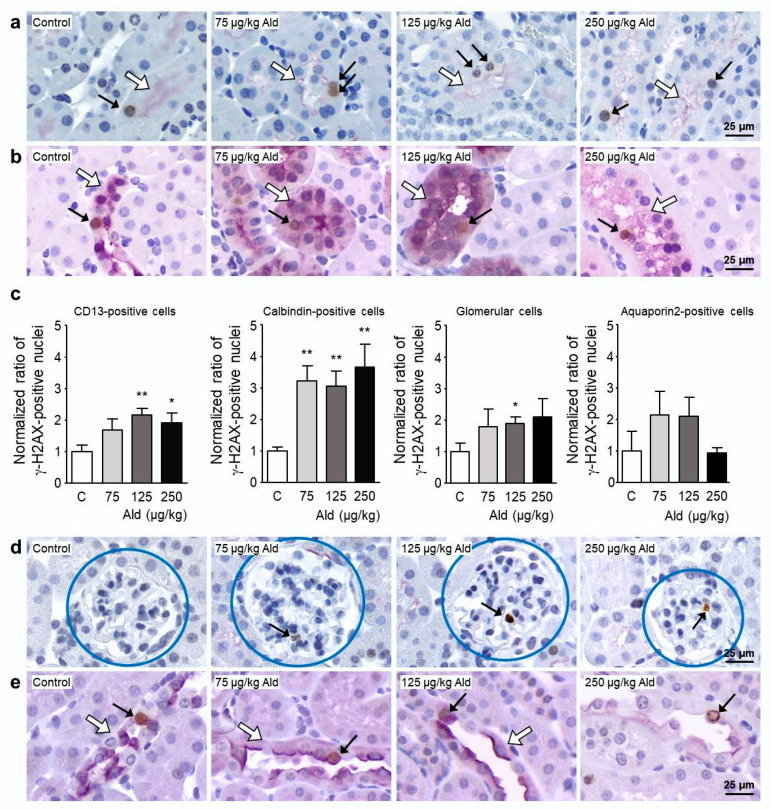
Localization of DNA damage caused by aldosterone infusion. (**a**,**b**,**d**,**e**) Representative images of those used for the localization of γ-H2AX in kidney cells. Double staining on paraffin-embedded kidney sections was carried out using antibodies against γ-H2AX (brown staining) and against cell-specific antigens (purple staining; (**a**) CD13, a marker specific for proximal tubule cells, located in the brush border; (**b**) calbindin, a marker specific for distal tubule cells and upper collecting duct cells, located in the cytosol; (**d**) glomeruli were identified due to their unique structure highlighted by the blue circles; (**e**) aquaporin 2, a marker for collecting duct cells and late distal tubular cells). Examples of γH2AX-positive stained nuclei are marked with black arrows, the staining specific for the kidney cell type is indicated with a white arrow. (**c**) Quantification of γ-H2AX-positive nuclei in the four kidney structures related to the number of nuclei in purple stained structures in 10 visual fields. For the quantification in the glomerulus, 50 glomeruli were evaluated. γ-H2AX: phosphorylated histone 2AX, Ald: aldosterone. Data are shown as mean + SEM, *n* = 5. * *p* ≤ 0.05, ** *p* < 0.01 vs. C: control group.

Examination of the localization of γ-H2AX-positive cells was conducted with the help of kidney cell specific antibodies, with CD13-positive cells belonging to proximal tubuli, calbindin-positive cells belonging to distal tubuli, and early collecting duct and aquaporin 2-positive cells belonging to late distal tubuli and collecting duct. The highest abundance of γ-H2AX staining was found in calbindin-positive cells, where a three-fold increase was quantified in all three dose groups whereas only a 1.5–2-fold increase could be found in CD13-positive, glomerular, and aquaporin 2-positive cells (Figure 5).

### 2.4. Abundance and Localization of Nrf2 in the Kidney

**Figure 6:**
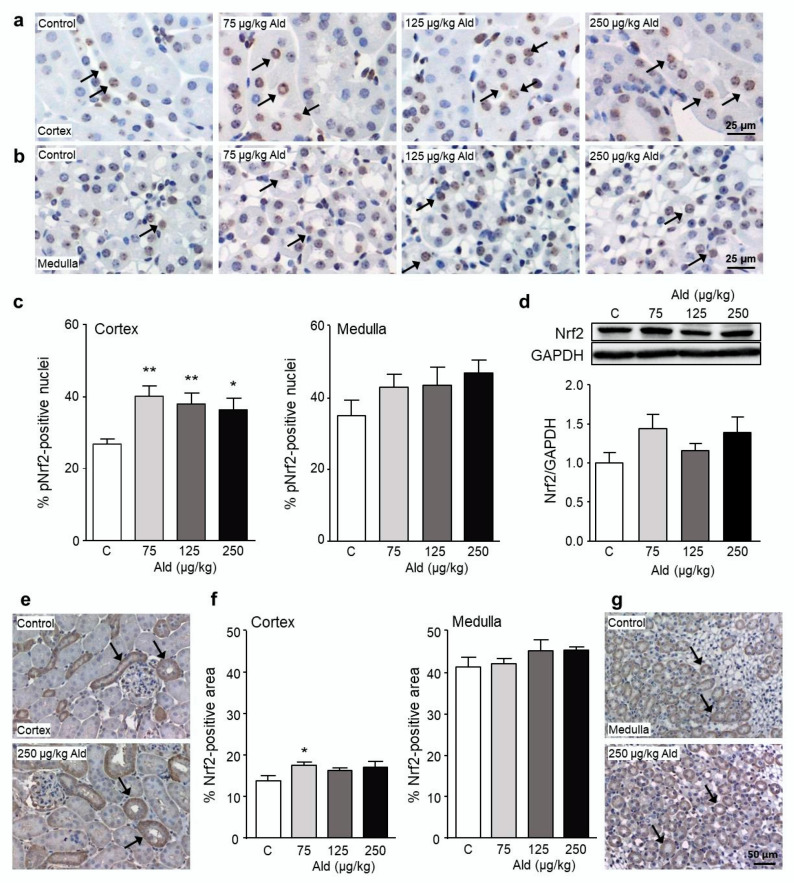
Induction of the transcription factor Nrf2 by aldosterone. Paraffin-embedded kidney sections were either stained with an antibody against Nrf2 phosphorylated at Ser40 representing activated Nrf2 (**a**–**c**) or with an antibody against unmodified Nrf2, representing total Nrf2 protein (**e**–**g**). Shown are representative pictures of cortical (**a/e**) and medullary (**b/g**) kidney sections from control and aldosterone-infused animals. Examples of positive stained nuclei (**a**,**b**) or areas (**e**,**g**) are marked with black arrows. Percentage of Nrf2-positive stained nuclei (**c**) and area (**f**). For the quantification of positive Nrf2 nuclei or area, 10 visual fields of cortical and 3–5 visual fields of medullary kidney sections were analyzed per animal via Image J. (**d**) Representative picture of the Western blots of the expression of total Nrf2 (95–110 kDa) in kidneys of mice as well as the quantification of band densities of the above-mentioned protein measured via Image J and related to the housekeeper GAPDH (37 kDa). Ald: aldosterone, GAPDH: glyceraldehyde 3-phosphate dehydrogenase, Nrf2: nuclear factor-erythoid-2-related factor 2. Data are shown as mean + SEM, *n* = 5. * *p* ≤ 0.05, ** *p* < 0.01 vs. C: control group.

Abundance of phosphorylated Nrf2 (phosphorylated at S40, pNrf2), a post-translational modification pointing to Nrf2 in an activated state, was first quantified in cortex and medulla of the kidney (Figure 6a–c). All dose groups, without an apparent dose-dependency, showed significantly increased pNrf2 in the cortex. In control animals more pNrf2-positive nuclei were found in the medulla than in the cortex, but with higher aldosterone doses only a tendential increase could be observed.

Non-phosphorylated total Nrf2 was tendentially but not significantly increased in kidney protein extracts from all treatment groups (Figure 6d). A quantification of total Nrf2 on kidney tissue also showed a tendential increase of protein amount in the cortex which was significant in the lowest dose group (Figure 6e,f). In the medulla, a higher basal amount of total Nrf2 was found compared to the cortex, which was not affected by aldosterone infusion. Non-phosphorylated Nrf2 was mainly present in the cytosol of tubuli whereas phosphorylated Nrf2 was only observed in nuclei, demonstrating the translocation of Nrf2 after phosphorylation at Ser40 (Figure 6a,e). Additionally, non-phosphorylated Nrf2 seems to be mainly expressed in distal tubuli as optically observed on the stained kidney sections (Figure 6f).

**Figure 7:**
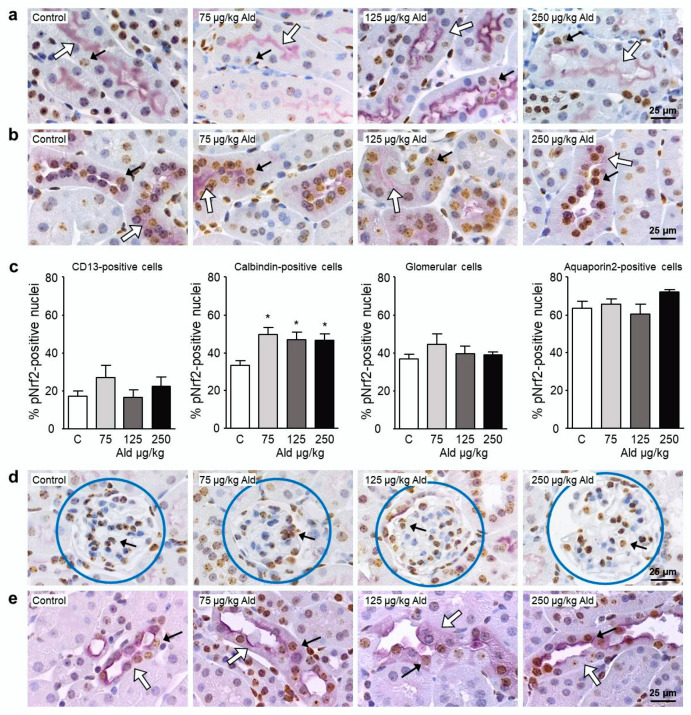
Localization of the activated transcription factor Nrf2. (**a**,**b**,**d**,**e**) Representative images of those used for the localization of pNrf2 in kidney cells. Double staining on paraffin-embedded kidney sections was carried out using antibodies against pNrf2 (brown staining) and against cell-specific antigens (purple staining; (**a**) CD13, a marker specific for proximal tubule cells, located in the brush border; (**b**) calbindin, a marker specific for distal tubule cells and upper collecting duct cells, located in the cytosol; (**d**) glomeruli were identified due to their unique structure highlighted by the blue circles; (**e**) aquaporin 2, a marker for collecting duct cells and late distal tubular cells). Examples of pNrf2-positive stained nuclei are marked with black arrows, the staining specific for the kidney cell type is indicated with a white arrow. (**c**) Quantification of pNrf2-positive nuclei in the four kidney structures related to the number of nuclei in purple stained structures in 10 visual fields. For the quantification in the glomerulus, 50 glomeruli were evaluated. Ald: aldosterone, Nrf2: nuclear factor-erythoid-2-related factor 2. Data are shown as mean + SEM, *n* = 5. * *p* ≤ 0.05 vs. C: control group.

When the distribution of pNrf2-positive nuclei among the four main kidney structures of the cortex, proximal tubuli, distal tubuli, glomeruli, and collecting ducts was studied, we first of all found a difference in the basal amount of pNrf2 in the four structures (Figure 7). While the basal level of pNrf2 in CD13-positive cells was around 20%, it was 50% higher in calbindin-positive cells, almost 100% higher in the glomeruli, and 200% higher in the aquaporin 2-positive cells. Aldosterone treatment led to a significant increase of pNrf2 abundance only in calbindin-positive cells, mainly representing distal tubuli (Figure 7c).

### 2.5. Effect of Aldosterone on Expression of Selected Genes and on Nrf2 Target Proteins

Despite causing an increase of nuclear-located pNrf2 in the kidney, aldosterone treatment did not increase the mRNA expression of Nrf2 target genes (Figure S3). On the contrary, most tested genes showed a tendency to be decreased, glutathione peroxidase 1 and NADPH quinone dehydrogenase 1 were even significantly lowered by the highest aldosterone dose. The mRNA of one of the physiological target genes of aldosterone, the α-subunit of the epithelial sodium channel (ENaC), was significantly higher expressed in all dose groups (Figure S3).

**Figure 8:**
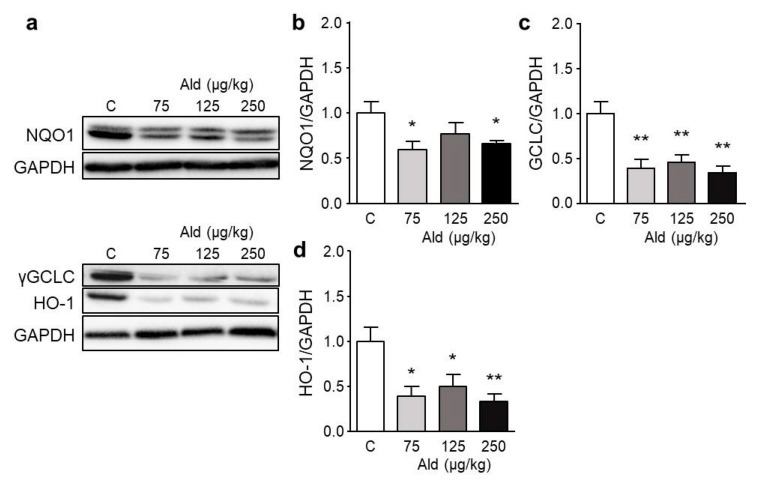
Expression of Nrf2-regulated proteins in aldosterone-infused mouse kidneys. (**a**) Representative pictures of Western blots of the expression of NQO1 (31 kDa), γGCLC (73 kDa), and HO1 (32 kDa) in kidneys of mice. Panels (**b**–**d**) show the quantification of band densities of the above-mentioned proteins measured via image J and related to the housekeeper GAPDH (37 kDa). Ald: aldosterone, GAPDH: glyceraldehyde 3-phosphate dehydrogenase, γGCLC: γ-glutamate cysteine ligase catalytic subunit, HO-1: heme oxygenase 1, NQO1: NADPH quinone dehydrogenase 1. Data are shown as mean + SEM, *n* = 5. * *p* ≤ 0.05, ** *p* < 0.01 vs. control group (C).

Further, aldosterone treatment led to a significant increase of the mRNA expression of the catalytic subunit of the pro-oxidative NADPH oxidase 2 and of the pro-inflammatory cytokine IL-6, while it had no effect on the mRNA expression of the pro-inflammatory cytokine IL-17A (Figure S3). The protein amounts of NADPH quinone dehydrogenase 1, γ-glutamate cysteine ligase, and heme oxygenase 1 were significantly reduced by all aldosterone doses while superoxide dismutase 1 and thioredoxin reductase 1 were not changed (Figure 8 and Figure S3). The amount of the heterodimerization partner of Nrf2, MafK which is indispensable for the DNA binding of Nrf2 was not changed significantly by aldosterone treatment (Figure S3).

Quantification of the Nrf2 target protein NADPH quinone dehydrogenase 1 on tissue slices revealed no significant change in the amount of protein, neither in the cortex nor in the medulla (Figure S4). 

## 3. Discussion

In kidneys of mice treated with aldosterone leading to a moderate kidney damage, we found a significant increase of phosphorylated Nrf2 and of oxidative damage in the form of DNA lesions, concentrated in distal tubuli.

Contrary to our expectations we could not measure a significantly elevated blood pressure induced by the used aldosterone concentrations. While there was a non-significant initial rise of blood pressure in all dose groups, an adaptation process seems to have started after 2 weeks of treatment, lowering the blood pressure in the end to control level. Three different aldosterone doses were tested here to find a dose which causes moderate but significant kidney damage within 28 days in mice. A literature search revealed a huge range of aldosterone concentrations used in mice (6.6–1000 µg/kg × d [23,24]), with concentrations from 100 µg/kg × d leading to significant blood pressure increases [25]. While none of the three chosen aldosterone concentrations increased the blood pressure robustly, already at the lowest concentration significant morphological and functional damage to the kidney could be observed. This damage was strictly dose dependent, but already the lowest dose resulted in a substantial increase in four out of five kidney function related parameters, suggesting that lower doses also would have the potential to damage the tissue. Comparable results, increased albumin excretion induced by aldosterone without elevated blood pressure, were published also by other groups [26,27].

While we were not able to find a significant increase in oxidative stress measured directly on kidney tissue neither with a redox-sensitive dye nor with an antibody against the oxidative base modification 8-oxodG or in urine in contrast to our previous studies with aldosterone- or angiotensin II-induced hypertension [20,28,29], we did find significantly increased excretion of the lipid peroxidation marker 15-isoprostane F_2t_. The only tendential increases of the markers could be due to the high interindividual variation found in the mice used in this pilot study. The animal number might have been too low to detect differences in these parameters which already intrinsically have a high variation. Nevertheless, the isoprostane and the surrogate marker of structural DNA damage, the phosphorylated histone 2AX (γ-H2AX), which in this kind of treatment can function as an indicator of oxidative stress, were significantly increased in the kidneys of aldosterone-infused mice. We reported aldosterone-induced DNA damage before in aldosterone-infused rats and in rats treated with the aldosterone precursor desoxycorticosterone acetate (DOCA) [20,22]. In the rat kidneys much more DNA damage was observed in the medulla, while here, in the mouse kidneys, DNA damage was only significantly increased in the cortex. When localizing the damage within the cortex, most cells with DNA damage were found in cells positive for the distal tubular cell/early collecting duct cell marker calbindin.

Activation of the master regulator of the antioxidative response, the transcription factor Nrf2, was investigated. To identify the active form of Nrf2, we decided to stain Nrf2 with an antibody recognizing Nrf2 phosphorylated at serine 40 (pNrf2). Additionally, we exclusively quantified Nrf2 localized in cell nuclei. Under stress-free conditions, Nrf2 is restrained in the cytoplasm in a complex with its prominent inhibitor Kelch-like ECH-associated protein 1 (Keap1), which promotes proteasomal degradation of Nrf2. Upon exposure of cells to ROS, modification of the cysteine residues in Keap1 releases Nrf2 to translocate into the nucleus [30]. There it forms heterodimers with small musculoaponeurotic fibrosarcoma (Maf) proteins and binds to the antioxidant responsive element (ARE) present in the promotor/enhancer regions of genes encoding antioxidant and detoxifying proteins [31]. Phosphorylation at serine 40 facilitates the dissociation from Keap1 and is involved in ARE transactivation [32,33]. pNrf2 was significantly increased by all aldosterone doses in the kidney cortex of mice. In rats, Nrf2 was increased in the whole kidney, with a higher abundance in the medulla, but detected with an antibody against the non-phosphorylated Nrf2 [21]. Within the cortex we found the highest amount of pNrf2 in cells positive for the distal tubular cell/early collecting duct cell marker calbindin. This is in accordance with other publications localizing Nrf2, its mRNA or one of its targets, heme oxygenase 1 in the kidney [34,35,36]. When quantifying total Nrf2, a tendential increase was found. In the calbindin-positive cells also the highest DNA damage was present. We could not find indicators of a diminished expression of DNA repair proteins in the aldosterone-treated animals. This leads to the question if the pNrf2 found in the nuclei of calbindin-positive cells was executing its function as a transcription factor. The effect of aldosterone on the actual activity of DNA repair proteins remains to be analyzed.

Studying Nrf2 targets either on the level of mRNA or protein expression within extracts of the whole kidney showed a tendential or a significant downregulation. It is known from CKD that Nrf2 and also Nrf2 targets like NQO1 are decreased in animal models and in patients [10,11]. In the other animal models we have studied ourselves up to now (angiotensin II-induced hypertension in mice, aldosterone-induced hypertension in rats, adenine-induced CKD) we, like in the present study, always found increased Nrf2 amounts [21,37,38]. In these models we also found an increase in at least one of the Nrf2 targets, heme oxygenase 1, superoxide dismutase, or γ-glutamate cysteine ligase. An explanation for the present results, where no Nrf2 target was induced, might be that although Nrf2 was phosphorylated and translocated into the nucleus it still might not be functional as a transcription factor. Phosphorylation and nuclear translocation are signs for an activation of Nrf2. However, for Nrf2 to regulate target gene expression a transcriptional complex formed out of various proteins at the promoter sequence is necessary, reviewed recently by Tonelli et al. [39]. Therefore, for example heterodimerization of Nrf2 with small Maf proteins is needed [40]. Since no change in the amount of one of the important dimerization partners MafK was detected, another mechanism possibly inhibits Nrf2 function like impairment of DNA binding by inhibitory factors like Bach1 or activating transcription factor 3 (ATF3) which compete for the binding sites on small Maf proteins or the DNA [41]. As the DNA damage is highest in the area of also high abundance of pNrf2, the observed Nrf2 activation is either not sufficient to protect the cells from oxidative damage or the pNrf2 is non-functional in these cells. This result, as well as the loss of kidney function in the aldosterone-induced kidney injury supports the need for a better understanding of Nrf2 effects during kidney damage and for effective Nrf2 activation by new Nrf2 activators to protect the kidney.

## 4. Materials and Methods

### 4.1. Animal Treatment

Twenty male C57BL/6-mice (Janvier, LE Genest Saint Isle, France) were randomly distributed into four equal-sized groups at the age of 10 weeks. Mice were equipped with osmotic mini pumps (Model 1004, Alzet, Durect Coporation, Cupertino, CA, USA) under inhalant isoflurane anesthesia (1.5–2%, anesthesia station MiniTAG, TEM SEGA, Pessac, France). The mini pumps delivered 75, 125, or 250 µg aldosterone/kg × day for 28 days. The control group received 15% EtOH in PBS as solvent control. Additionally, all mice had free access to food and 1% (*w*/*v*) NaCl as drinking water. Preemptive analgesia was provided with 5 mg/kg carprofen (Zoetis Deutschland GmbH, Berlin, Germany). The blood pressure was measured twice weekly non-invasively via the tail cuff method (Visitech Systems, Apex, NC, USA). The mice were adapted to the blood pressure measurement procedure 2 weeks before the implantation of mini pumps. At the beginning and the end of the experiment mice were placed into metabolic cages for 23 h to obtain urine samples. After 4 weeks of treatment, mice were anesthetized deeply (120 mg ketamine/kg and 8 mg xylazine/kg i.m.) and perfused with ice-cold Deltadex 40 (AlleMan Pharma GmbH, Rimbach, Germany) containing 1% procainhydrochloride (bela-pharm, Vechta, Germany), followed by ice-cold 0.9% NaCl solution (Fresenius Kabi Deutschland GmbH, Bad Homburg, Germany). Kidneys and hearts were isolated, weighed, and either embedded in paraffin or shock-frozen in liquid nitrogen and stored at −80 °C.

### 4.2. Quantification of Aldosterone

Serum levels of aldosterone were measured using the Aldosterone ELISA Kit (BT E-5200, BioTrend, Cologne, Germany) according to the manufacturer’s instructions.

### 4.3. Parameters of Renal Function

Renal function was assessed by calculating the creatinine clearance, measuring albumin and neutrophil gelatinase-associated lipocalin (NGAL) excretion. Urinary and serum creatinine was quantified with Creatinine urinary/serum Colorimetric Assay Kits (No. 500701/700460, Cayman Chemical Company, Ann Arbor, MI, USA) according to the protocol provided by the manufacturer. For the quantification of albumin and NGAL excretion, the Mouse Albumin ELISA Kit (EMA3201-1. Assay Pro, St. Charles, IL, USA) and the Mouse NGAL ELISA Kit (KIT 042, BioPorto, Gentofte, Denmark) were used according to the manufacturer’s protocol. Albumin and NGAL were related to urinary creatinine.

### 4.4. Histopathology

For histopathological investigations of the kidney, 3 µm paraffin sections were stained with hematoxylin and eosin (HE), periodic acid–Schiff stain (PAS), and Sirius red (SR). Tubular as well as glomerular damage was determined as reported earlier [42]. 

### 4.5. Immunohistochemistry

Kidney sections (3 µm) (RM 2164, Leica, Wetzlar, Germany) were mounted on glass slides, heated for 1 h at 60 °C, and deparaffinized with Roti-Histol (Roth, Karlsruhe, Germany) and ethanol. Antigen retrieval was performed with citrate buffer (DAKO Retrieval Solution, pH 6.0, Agilent Technologies, Santa Clara, CA, USA) at 95 °C for 30 min. Afterwards the slides were blocked and incubated with the corresponding primary antibodies at 4 °C overnight. The specific antibodies and dilutions were as follows: anti-γ-H2AX (#9718, 1:200, Cell Signaling, Herts, UK), anti-Nrf2 (sc-722, 1:1000, Santa Cruz Biotechnology, Dallas, TX, USA), anti-pNrf2 (phospho S40, ab76026, 1:1000, abcam, Cambridge, UK), anti-NQO1 (ab34173, 1:500, abcam, Cambridge, UK), and anti-PCNA (MAB424R, 1:1000, Merck, Darmstadt, Germany). Subsequently, the sections were incubated with the biotinylated secondary antibodies goat anti-rabbit (ab6720, 1:200, abcam, Cambridge, UK) or goat anti-mouse (ab6788, 1:200, abcam, Cambridge, UK) for 45 min at room temperature. Antibody binding was visualized as described before [21,28]. Sections were counterstained with hematoxylin. Pictures were taken at 200-fold magnification. The ratio of positive to negative nuclei or area was assessed via Image J (http://imagej.nih.gov/ij/index.html) within 10–15 visual fields of the cortex and 3–5 visual fields of the medulla.

### 4.6. Double Staining

For the localization of positive γ-H2AX and pNrf2 in the kidney a double staining was performed identifying γ-H2AX or pNrf2 positive staining within different types of kidney cells. The staining of the first antigen (γ-H2AX or pNrf2) was carried out as described above using diaminobenzidine (DAB) as chromogen. After visualization of the antibody binding the protocol was repeated using different primary antibodies for identifying different cell types of the kidney. For the identification of proximal tubular cells an antibody against CD13 (aminopeptidase N, ab108310, abcam, Cambridge, UK) was diluted 1:6000 in PBS. Distal tubular cells and cells of the early collecting duct were identified using an antibody against calbindin (1:200, #2173, Cell Signaling, Herts, UK). Late distal tubular cells and collecting duct cells were identified with an antibody against aquaporin 2 (1:500, ab15116, abcam, Cambridge, UK). For visualizing the second antigen the VECTOR^®^ VIP Peroxidase Substrate Kit was used (SK-4600, Vector Lab, Burlingame, CA, USA) resulting in a purple staining. Sections were counterstained with hematoxylin and dehydrated skipping steps with lower grades of ethanol. Pictures were taken at 400-fold magnification. Glomeruli were identified by their structure and 50 glomeruli were analyzed per animal. The ratio of positive to negative nuclei for γ-H2AX or pNrf2 on 10 visual fields was assessed via Image J (http://imagej.nih.gov/ij/index.html) by counting only nuclei positive for the specific kidney marker. 

### 4.7. Detection of Reactive Oxygen Species

Reactive oxygen species (ROS) production was measured by staining 8 µm cryosection with 10 µM dihydroethidium (DHE) for 20 min as described previously [22]. 

### 4.8. Quantification of 8-oxodG in Kidneys and 8-OHdG in Urine

Kidney sections (3 µm) treated as described above were stained for 8-oxodG as reported before with slight modifications [21]. Non-specific binding was blocked sequentially with 3% bovine serum albumin (BSA) and TNB-Blocking buffer (Perkin Elmer, Waltham, MA, USA) for 30 min each at 37 °C. The primary antibody against 8-oxodG (Immundiagnostik AG, Bensheim, Germany, #AA1005.2) was applied 1:1000 in 3% BSA and incubated at 4 °C overnight, followed by an incubation with a secondary antibody (biotinylated goat anti-mouse 1:200) for 45 min. For signal amplification the Tyramide Signal Amplification Biotin System (NEK700A001kit, Perkin Elmer, Waltham, MA, USA) was used according to the manufacturer’s instructions. Antibody binding was visualized with a diaminobenzidine kit (SK-4100, Vector Lab, Burlingame, CA, USA). Pictures were taken at a 200-fold magnification. The ratio of positive to negative nuclei was assessed via Image J (http://imagej.nih.gov/ij/index.html) within 10 visual fields of the cortex and five visual fields of the medulla. 8-hydroxy-2′-deoxyguanosine in urine was quantified with the DNA damage (8-OHdG) ELISA Kit from StressMarq (StessMarq Biosciences Inc., Victoria, BC, Canada) according to the protocol provided by the manufacturer.

### 4.9. Quantification of 15-Isoprostane F_2t_ in Urine

The level of 15-isoprostane F_2t_ in urine was determined with the Urinary Isoprostane ELISA Kit (EA85, Oxford Biomedical Research, Rochester Hills, MI, USA) according to the manufacturer’s protocol. 15-isoprostane F_2t_ was related to urinary creatinine.

### 4.10. Western Blot

For protein isolation frozen kidney tissue was manually pestled and lysed in RIPA buffer (50 mM Tris, 150 mM NaCl, 1 mM EDTA, 0.025% Natriumdesoxycholat, 1% Nonidet, 1 mM NaF) supplemented with a protease inhibitor cocktail (Sigma, Taufkirchen, Germany) and a phosphatase inhibitor cocktail (Thermo Scientific, Rockford, IL, USA). After a centrifugation step at 10,000× *g* for 15 min at 4 °C the protein extracts were stored at −80 °C. Then, 50 µg protein was loaded on a SDS gel and after separation transferred to a nitrocellulose membrane (GE Healthcare, Little Chalfont, UK). Membranes were incubated with specific primary antibodies against GCLC (PA1492, BosterBio, Pleasanton, CA, USA), HO-1 (ab13243, abcam, Cambridge, UK), MafK (GTX129240, GeneTex, Irvine, CA, USA), NQO1 (ab34173, abcam, Cambridge, UK), Nrf2 (sc-722, Santa Cruz Biotechnology, Dallas, TX, USA), PARP (#9542, Cell Signaling, Herts, UK), pp47 phox (phospho S328, orb256707, Biorbyt, Cambridge, UK), SOD1 (GTX100554, GeneTex, Irvine, CA, USA), TrxR1 (GTX108727, GeneTex, Irvine, CA, USA), and GAPDH (#2118, Cell Signaling, Herts, UK) overnight at 4 °C, followed by an incubation with HRP-conjugated secondary antibodies for 2.5 h at room temperature. Antibody binding was visualized using the BM Chemiluminescence Blotting Substrate Kit (Roche, Basel, Switzerland) according to the manufacturer’s instructions. Chemiluminescence signals were recorded using the ChemiDoc™ Touch Imaging System (BIO-RAD, Hercules, CA, USA).

### 4.11. Quantitative RT-PCR

mRNA was isolated from 20–40 mg frozen kidney tissue using the QIAcube (Qiagen, Hilden Germany) according to the manufacturer’s instructions. Isolated mRNA was reverse transcribed to cDNA using the High Capacity cDNA Reverse Transcription Kit (Thermo Fisher, Waltham, MA, USA). Quantitative Real-Time PCR (qRT-PCR) was performed with 20 µg cDNA and the SensiMix SYBR Hi-ROX Mastermix (Bioline GmbH, Luckenwalde, Germany) using the CFX96 Real-Time System (BIO-RAD, Hercules, CA, USA). Primer sequences used for gene expression analysis are listed in Table S2. Relative expression levels of target genes were normalized to the housekeepers GAPDH and β-actin and calculated using the comparative CT method with the analysis software Bio-Rad CFX Manager 3.1 (BIO-RAD, Hercules, CA, USA).

### 4.12. Statistics

The data from four to five animals per group are shown as mean ± standard error mean (SEM). For statistical analyses GraphPad Prism 6 (GraphPad Software, La Jolla, CA, USA) was used. The data were tested for a normal distribution with the use of the Kolmogorov–Smirnov test with Dallal–Wilkinson–Liliefor *p* values. Differences between the control group and the aldosterone groups were tested for significance using the unpaired two-tailed Student’s *t* test (normal distribution) or the Mann–Whitney U test (non-normal distribution). A *p* value ≤ 0.05 was considered significant.

## 5. Conclusions

In a model of moderate kidney damage induced by aldosterone-infusion, increased phosphorylation of the master regulator of the antioxidative response, Nrf2, was found. Localization of the phosphorylated Nrf2 revealed an increase mainly in distal tubuli. Here, also the highest DNA damage, representative for oxidative damage to the cells, was located. Surprisingly, no higher expression of Nrf2 targets could be detected in the kidneys, hinting to a yet to be identified inhibitory post-Nrf2 phosphorylation mechanism induced by aldosterone-mediated kidney damage.

## Figures and Tables

**Table 1:** Body weight, body weight ratios, and parameters of kidney function after 28 days of aldosterone infusion. Ald: aldosterone, NGAL: neutrophil gelatinase-associated lipocalin. Data are shown as mean ± SEM. * *p* ≤ 0.05, ** *p* < 0.01, *** *p* < 0.001 vs. control group.

Parameter	Control	75 µg/kg Ald	125 µg/kg Ald	250 µg/kg Ald
Body weight (g)	27.3 ± 0.4	27.7 ± 0.3	27.1 ± 0.9	26.0 ± 0.8
Kidney/body weight (‰)	6.3 ± 0.2	8.7 ± 0.4 ***	9.6 ± 0.3 ***	9.7 ± 0.2 ***
Heart/body weight (‰)	4.9 ± 0.1	5.8 ± 0.5	5.7 ± 0.2 *	5.5 ± 0.1 **
Drinking volume (mL/23 h)	7.6 ± 1.7	16.8 ± 2.1 **	19.7 ± 5.9	12.7 ± 5.2
Diuresis (mL/23 h)	3.5 ± 2.3	9.9 ± 2.4	12.0 ± 5.2	9.3 ± 3.9
Creatinine clearance (mL/h)	2.4 ± 0.5	4.0 ± 0.8	4.6 ± 1.0	3.0 ± 0.4
Albumin/creatinine (µg/mg)	111 ± 15	1776 ± 678 *	2520 ± 1563 *	2716 ± 913 *
NGAL/creatinine (ng/mg)	138 ± 30	541 ± 70 **	817 ± 156 *	1087 ± 221 **

**Table 2:** Quantification of morphological changes. For every staining 18 visual fields or 30 glomeruli per animal were analyzed under the microscope. Ald: aldosterone, GSI: glomerular sclerosis index, MSI: mesangiolysis index. Data are shown as mean ± SEM, *n* = 4–5. * *p* ≤ 0.05, ** *p* < 0.01, *** *p* < 0.001 vs. control group.

Parameter		Control	75 µg/kg Ald	125 µg/kg Ald	250 µg/kg Ald
Inflamed area (%)		0.5 ± 0.1	0.4 ± 0.1	1.61 ± 0.5*	1.9 ± 0.6 *
Atrophic basal membranes (%)		4.7 ± 1.8	20.2 ± 6.0 *	23.9 ± 7.5 **	27.3 ± 5.0 **
Collagen deposition (%)		1.9 ± 0.7	7.5 ± 1.5 **	10.9 ± 1.7 **	11.7 ± 1.7 ***
Glomerular damage	GSI	0.58 ± 0.10	1.18 ± 0.12 **	1.32 ± 0.08 ***	0.97 ± 0.11 *
	MSI	0.57 ± 0.11	0.84 ± 0.05	0.83 ± 0.02	0.93 ± 0.09 **

## Supplementary Materials

Can be found at https://www.mdpi.com/1422-0067/21/13/4679/s1.

## Abbreviations

8-OHdG	8-hydroxy-2′-deoxyguanosine
8-oxodG	8-oxo-2′-deoxyguanosine
αENaC	α-subunit of the epithelial sodium channel
γ-H2AX	phosphorylated histone 2AX
ACEi	angiotensin converting enzyme inhibitors
Ald	aldosterone
Apex1	apurinic/apyrimidinic endonuclease 1
ARB	angiotensin II receptor blockers
ARE	antioxidant responsive element
ATF3	activating transcription factor 3
Atm	ataxia telangiectasia mutated homologue
Brca1	breast cancer 1
CD	cluster of differentiation
CKD	chronic kidney disease
DHE	dihydroethidium
DOCA	desoxycorticosterone acetate
EDTA	ethylenediaminetetraacetic acid
GAPDH	glyceraldehyde 3-phosphate dehydrogenase
GCLC	γ-glutamate cysteine ligase catalytic subunit
GCLM	γ-glutamate-cysteine ligase modifier subunit
GPx1	glutathione peroxidase 1
HE	hematoxylin and eosin stain
HO-1	heme oxygenase 1
IL	interleukin
Keap1	Kelch-like ECH-associated protein 1
Lig	ligase
Maf	musculoaponeurotic fibrosarcoma
NADPH	reduced nicotinamide adenine dinucleotide phosphate
NF-κB	nuclear factor ‘kappa-light-chain-enhancer’ of activated B-cells
NGAL	neutrophil gelatinase-associated lipocalin
Nox2	NADPH oxidase 2 subunit p90
NQO1	NADPH quinone dehydrogenase 1
Nrf2	nuclear factor-erythroid-2-related factor 2
Ogg1	8-oxoguanine DNA-glucosylase 1
PARP	poly (ADP-ribose) polymerase
PAS	periodic acid–Schiff stain
PCNA	proliferating cell nuclear antigen
pNrf2	phosphorylated nuclear factor-erythroid-2-related factor 2
pp47phox	phosphorylated NADPH oxidase 2 activator
RAAS	renin-angiotensin-aldosterone system
ROS	reactive oxygen species
SEM	standard error mean
SOD1	superoxide dismutase 1
SR	Sirius red stain
TGF-β	transforming growth factor-β
TNF-α	tumor necrosis factor-α
TrxR1	thioredoxin reductase 1
