# Supplementary material for: Aldosterone Induces DNA Damage and Activation of Nrf2 Mainly in Tubuli of Mouse Kidneys
Source: Int J Mol Sci. 2020 Jun 30;21(13):4679. doi: 10.3390/ijms21134679 (PMC7370281; doi:10.3390/ijms21134679)
Supplement: Supplementary file 1 [file ijms-21-04679-s001.pdf]

International Journal of *Molecular Sciences*

Article

Aldosterone induces DNA damage and activation of Nrf 2 mainly in tubuli of mouse kidneys

Ronja Balhorn [1], Christina Hartmann [1] and Nicole Schupp [1,*]

[1] Institute of Toxicology, University of Düsseldorf, 40225 Düsseldorf, Germany; Balhorn.Ronja@hhu.de (R.B.); Christina.Hartmann.2@hhu.de (C.H.)
* Correspondence: schupp@hhu.de, Tel.: +49-211-8113001

Received: date; Accepted: date; Published: date

Supplemental material

Table S1. Cytokines measured in serum of control and aldosterone-infused mice with the help of the LEGENDplex™ inflammation-panel. The serum levels of the shown cytokines IL-6, IL-10, IL-12p70, IL-17A, IL-23 and IL-27 were normalized to the control group. Ald: aldosterone, IL: interleukin. Data are shown as mean + SEM, n=4-5. *p≤0.05 vs. control group.

Parameter	Control	75 µg/kg Ald	125 µg/kg Ald	250 µg/kg Ald
IL-6	1.00 ± 0.28	0.96 ± 0.18	0.79 ± 0.15	1.49 ± 0.34
IL-10	1.00 ± 0.29	2.50 ± 1.01	0.90 ± 0.32	2.02 ± 0.69
IL-12p70	1.00 ± 0.55	0.05 ± 0.00	0.05 ± 0.00	1.03 ± 0.48
IL-17A	1.00 ± 0.31	0.43 ± 0.20	0.11 ± 0.06*	0.39 ± 0.12
IL-23	1.00 ± 0.85	0.35 ± 0.05	0.16 ± 0.07	0.78 ± 0.20
IL-27	1.00 ± 0.45	1.81 ±0.60	0.64 ± 0.25	5.52 ± 3.16

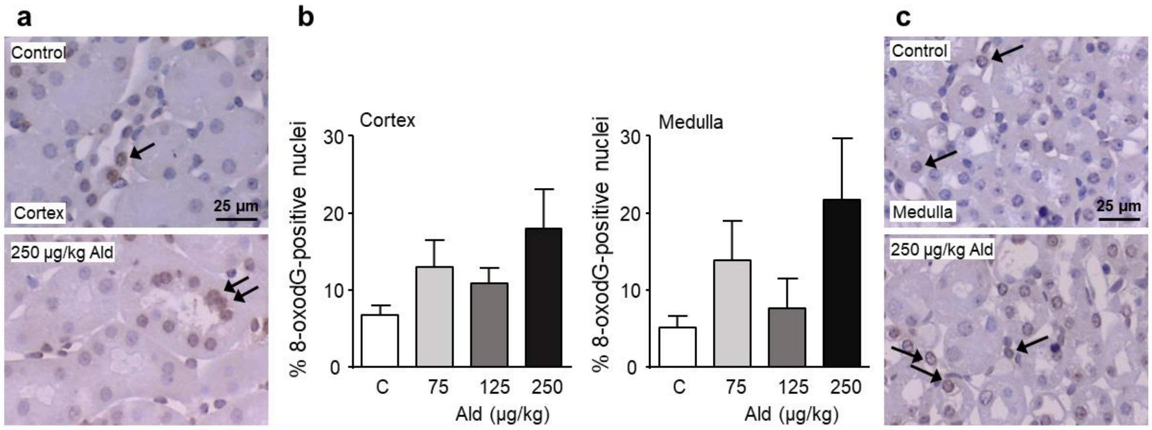

Figure S1. Oxidative DNA base damage caused by aldosterone infusion. Paraffin-embedded kidney sections were stained with an antibody against 8-oxodG, a marker of oxdative DNA damage. Staining of the oxidative base modification 8-oxodG in cortex (**a**) and medulla (**c**) and quantification of the percentage of positive nuclei (**b**). For the quantification of 8-oxodG-positive nuclei, 10 visual fields of cortical and 5 visual fields of medullary kidney sections were analyzed per animal via Image J. Examples of positive stained nuclei are marked with black arrows. 8-oxodG: 8-oxo-2'-deoxyguanosine, Ald: aldosterone. Data are shown as mean + SEM, n=5.

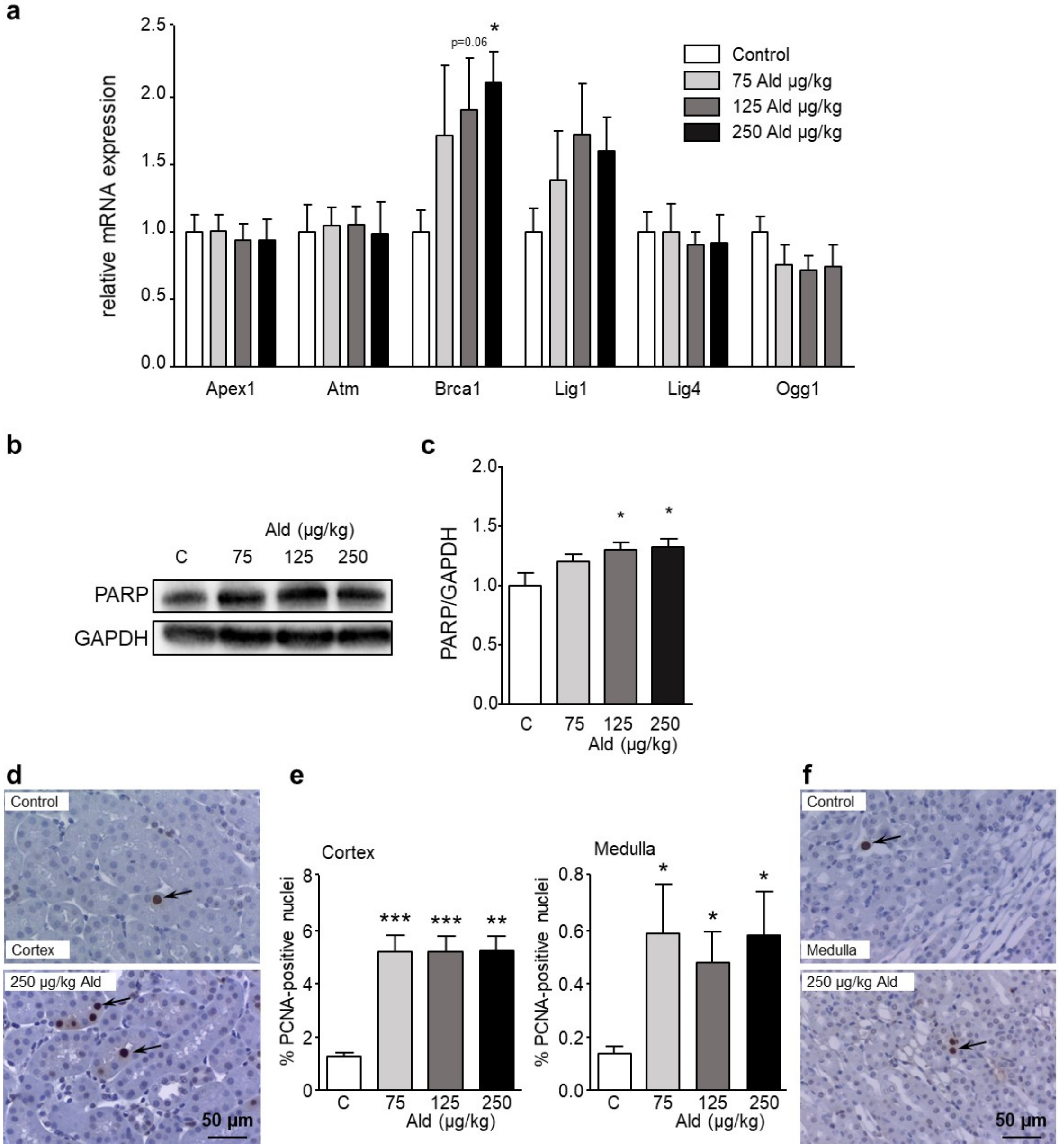

Figure S2. Expression of DNA damage response related genes and proteins in aldosterone infused mice. (**a**) The mRNA expression of *Apex1*, *Atm*, *Brac1*, *Lig1*, *Lig4* and *Ogg1* in kidneys of mice was evaluated via RT-PCR. RNA was isolated from kidneys of control and aldosterone infused mice. mRNA levels were referred to *GAPDH* and *β-actin* as housekeeper genes. Representative picture of the western blots of the expression of PARP (113 kDa) in kidneys of mice (**b**). (**c**) Shown is the quantification of band densities of the above mentioned protein measured via image J and related to the housekeeper GAPDH (37 kDa). Paraffin-embedded kidney sections were stained with an antibody against PCNA. Shown are representative pictures of cortex (**d**) and medulla (**f**) and quantification of the percentage of PCNA-positive nuclei (**e**). For the quantification of PCNA-positive nuclei, 10 visual fields of cortical and 5 visual fields of medullary kidney sections were analyzed per animal via Image J. Examples of positive stained nuclei are marked with black arrows. Data are shown as mean + SEM, n=5. Ald: aldosterone, Apex1: apurinic/apyrimidinic endonuclease 1, Atm: ataxia telangiectasia mutated homologue, Brca1: breast cancer 1, GAPDH: glyceraldehyde 3-phosphate dehydrogenase, Lig1: ligase 1, Lig4: ligase 4, Ogg1: 8-oxoguanine DNA-glycosylase 1, GAPDH: glyceraldehyde 3-phosphate dehydrogenase, PARP: Poly (ADP-ribose) polymerase, PCNA : proliferating cell nuclear antigen. *p≤0.05, **p<0.01, ***p<0.001 vs. C: control group.

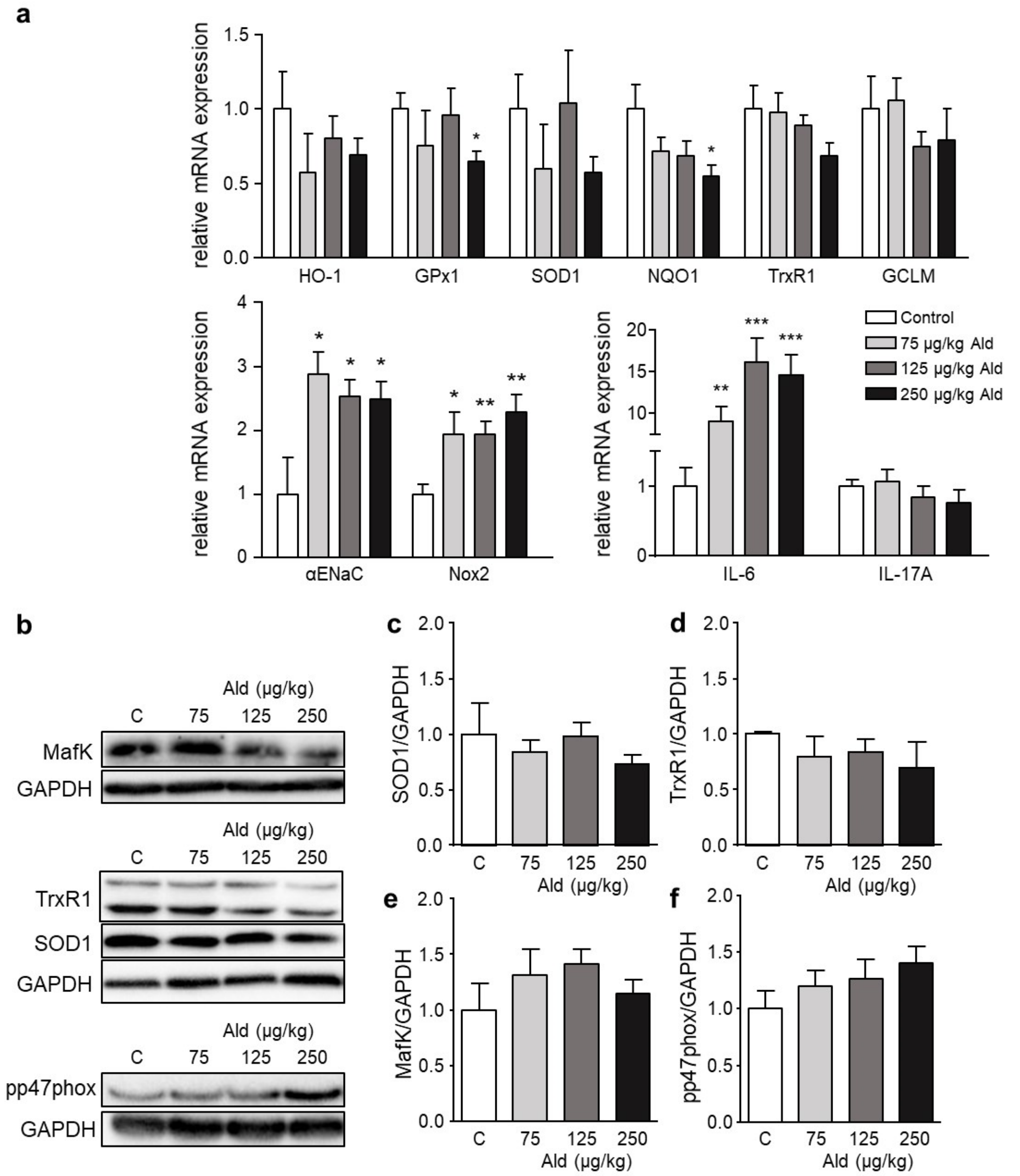

Figure S3. Expression of aldosterone- and Nrf2-regulated genes and proteins in aldosterone infused mouse kidneys. (**a**) mRNA expression of the indicated genes in kidneys of mice was evaluated via qRT-PCR. mRNA levels were measured and referred to *GAPDH* and *β-actin* as housekeeper genes. (**b**) Representative pictures of western blots of the expression of SOD1 (16 kDa), TrxR1 (71 kDa), MafK (18 kDa) and pp47phox (47 kDa) in kidneys of mice. Panels (**c-f**) show the quantification of band densities of the above mentioned proteins measured via image J and related to the housekeeper GAPDH (37 kDa). Ald: aldosterone, GAPDH: glyceraldehyde 3-phosphate dehydrogenase, GCLM: γ-glutamate-cysteine ligase modifier subunit, GPx1: glutathione peroxidase 1, αENaC: α-subunit of the epithelial sodium channel, HO-1: heme oxygenase 1, IL-6: interleukin 6, IL-17A: interleukin 17A, MafK: musculoaponeurotic fibrosarcoma K, Nox2: NADPH oxidase 2 subunit p90, NQO1: NADPH quinone dehydrogenase 1, pp47phox: phosphorylated NADPH oxidase 2 activator, SOD1: superoxide dismutase 1, TrxR1: thioredoxin reductase 1. Data are shown as mean + SEM, n=5. *p≤0.05, **p<0.01, ***p<0.001 vs. C: control group.

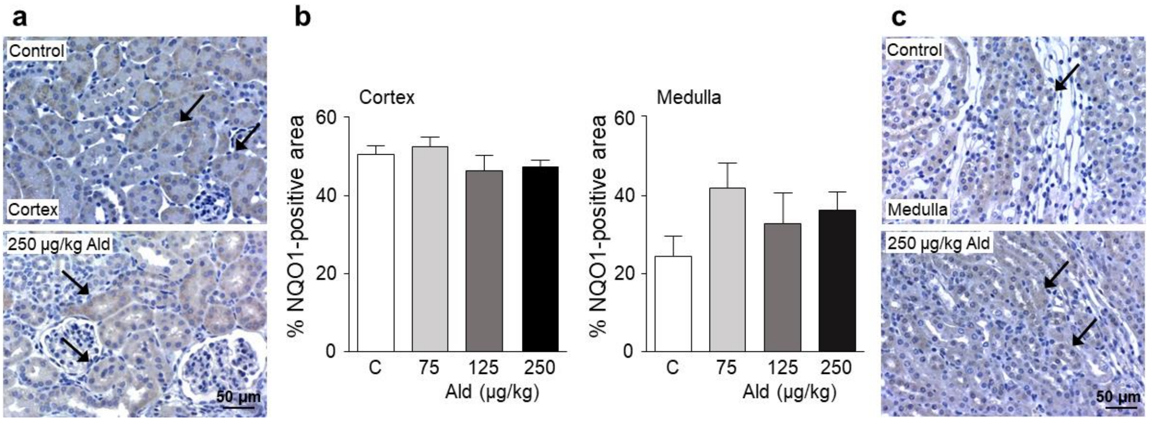

Figure S4. Expression of the Nrf2 target NQO1 in kidney tissue after aldosterone treatment. (**a**) Paraffin-embedded kidney sections were stained with an antibody against NQO1. Shown are representative pictures of cortex (**a**) and medulla (**c**) and quantification of the percentage of NQO1-positive area (**b**). For the quantification of NQO1-positive area, 15 visual fields of cortical and 5 visual fields of medullary kidney sections were analyzed per animal via Image J. Examples of positive stained areas are marked with black arrows. Ald: aldosterone. NQO1: NADPH quinone dehydrogenase 1. Data are shown as mean + SEM, n=5.

Table S2: Primers used for qRT-PCR

**Gene**	**Locus**	**Forward (5´-)**	**Reverse (5´-)**
ß-actin	NM_007393	GCATTGCTGACAGGATGCAG	CCTGCTTGCTGATCCACATC
αENaC	NM_011324	TGCACCCTTAATCCTTACAGATACA	CCTGGCGAGTGTAGGAAGAG
Apex1	NM_009687	AGAAATTGACCTCCGTAACC	CGCCAACCAACATTCTTAGA
Atm	NM_007499	ACCAGAGGATGCTGTTCA	ATCATTAAGTCTATGTTGAGTCCAA
Brca1	NM_009764	TTGTGAGCGTTTGAATGA	ACCTGGCTTAGTTACTGT
Gapdh	NM_008084	TCTCCTGCGACTTCAACA	TCTCTTGCTCAGTGTCCTT
Gclm	NM_008129	TTCTCGGGTGAGGTTTCTGC	AACGAGGGAGCTGTTTCCTG
Gpx1	NM_008160	TTGGTGATTACTGGCTGC	TGATATTCAGCACTTTATTCTTAGTAG
Ho-1	NM_010442	CCAGAGTCCCTCACAGAT	CCCAAGAGAAGAGAGCCA
IL-6	NM_031168	AGTTGCCTTCTTGGGACTGA	CAGAATTGCCATTGCACAAC
IL-10	NM_010548	CATGGGTCTTGGGAAGAGAA	CATTCCCAGAGGAATTGCAT
IL-17A	NM_010552	TCTCCACCGCAATGAAGACC	AAAGTGAAGGGGCAGCTCTC
Lig1	NM_010715	ATTTCGGGTTTGCGTCTC	ACCACTTGATTCCTCTCCTT
Lig4	NM_176953	GTGTCCTGATGCTTAGTTGT	CTCCTTGAAGTGCCTGATT
Nox2	NM_007807	GCGGTGTGCAGTGCTATCAT	GGTTCCAGTGCGTGTTGCT
Nqo1	NM_008706	GGCCGATTCAGAGTGGCAT	CCAGACGGTTTCCAGACGTT
Ogg1	NM_010957	TGAGACTGCTGAGACAAGA	GGAAGCCATGATAAGTGACA
Sod1	NM_011434	ACCAGTTGTGTTGTCAGG	TTTCTTAGAGTGAGGATTAAAATGAG
TrxR1	NM_015762	CAGTTCGTCCCAACGAAAAT	GCACATTGGTCTGCTCTTCA

© 2020 by the authors. Licensee MDPI, Basel, Switzerland. This article is an open access article distributed under the terms and conditions of the Creative Commons Attribution (CC BY) license (http://creativecommons.org/licenses/by/4.0/).
